# Adsorption of Anionic Dye on the Acid-Functionalized Bentonite

**DOI:** 10.3390/ma13163600

**Published:** 2020-08-14

**Authors:** Jucielle Veras Fernandes, Alisson Mendes Rodrigues, Romualdo Rodrigues Menezes, Gelmires de Araújo Neves

**Affiliations:** 1 Programa de Pós-Graduação em Ciência e Engenharia de Materiais (PPG-CEMat), Universidade Federal de Campina Grande, Av. Aprígio Veloso-882, Bodocongó, Campina Grande-PB 58429-900, Brazil; alisson.mendes@professor.ufcg.edu.br (A.M.R.); romualdo.menezes@ufcg.edu.br (R.R.M.); gelmires.neves@ufcg.edu.br (G.d.A.N.); 2 Unidade Acadêmica de Engenharia de Materiais, Centro de Ciência e Tecnologia, Universidade Federal de Campina Grande, Av. Aprígio Veloso-882, Bodocongó, Campina Grande-PB 58429-900, Brazil

**Keywords:** bentonite, methylene blue, methyl orange, acid functionalization, adsorptive capacity

## Abstract

The efficiency of acid treatment on natural calcium bentonite (natural bentonite) for anionic dye adsorption was investigated using methyl orange (MO) as a probe. Additionally, adsorption experiments were accomplished between the natural bentonite, acidified bentonite, and a cationic dye (methylene blue, MB). Acid functionalization in natural bentonite (RF) was carried out with HCl and H_2_SO_4_ acids (RF1 and RF2, respectively). The samples were characterized by chemical analysis, mineralogy, particle size, and thermal behavior with the associated mass losses. The adsorption efficiency of MO and MB dyes was investigated by the effects of the initial concentration of adsorbate ($C_{i}$) and the contact time ($t_{c}$). The acid treatment was efficient for increasing the adsorption capacity of the anionic dye, and the $Q_{max}^{exp}$ values measured were 2.2 mg/g, 67.4 mg/g e 47.8 mg/g to RF, RF1 e RF2, respectively. On the other hand, the acid functionalization of bentonite did not significantly modify the MB dye adsorption. The Sips equation was the best fit for the adsorption isotherms. Thus, we found that the acid-functionalized bentonite increases the anionic dye adsorption by up to 8000%. The increased adsorptive capacity of acidified bentonite was explained in terms of electrostatic attraction between the clay surface and the dye molecule.

## 1. Introduction

Synthetic dyes are polluting agents with complex, non-biodegradable chemical structures that cause harmful effects both for the aquatic life and for human beings [1]. These dyes can be classified as anionic, cationic, and non-ionic [2]. Anionic dyes, when in aqueous solution, have a negative charge, such as those of sulfonate groups (SO^3−^). In turn, the cationic dyes (due to chemical groups such as the amine or sulfur) have a net positive charge [3]. The methyl orange dye (anionic dye) has high toxicity, a mutagenic and carcinogenic nature [4], a hard-breaking molecular structure [5], and difficult adsorption in different materials. On the other hand, methylene blue (cationic dye) does not have high toxicity but may cause harmful effects on human health, such as vomiting, increased heart rate, diarrhea, and cyanosis [6]. Disposing of both dyes in wastewaters in streams and aquifers may hamper reoxygenation and prevent aquatic life photosynthesis [7,8,9].

The removal of such dyes from wastewaters is often conducted via membrane filtration, ion-exchange separation, degradation, flotation, chemical oxidation, electrochemical treatment, adsorption [10,11,12,13]. Among these processes, adsorption shows more significant economic potential for removing dyes due to its efficiency, low energy consumption, and easy operation [14,15]. Different types of adsorbents can be used to remove contaminants, such as activated charcoal [16,17,18,19], polymeric [20,21,22,23], and biological materials [24,25,26,27,28]. These adsorbents have a high capacity for removing dyes, but their high cost and low capacity for reuse restrict their use [29]. Therefore, the search for low-cost adsorbents, such as clay minerals, has been extensively studied [30].

From the literature, it is possible to find several studies about the MO dye sorption in different adsorbents. Among them, the study published by Sabarish and Unnikrishnan stands out [31], wherein the zeolite hybrid matrix membranes (PVA/PDADMAC/ZSM-5) were used, and the $Q_{max}^{exp}$ obtained was equal to 76.4 mg/g. The zeolite incorporation in the PVA/PDADMAC/ZSM-5 makes the membrane surface heterogeneous, promoting this material as an alternative for removing MO from industrial streams. Robati et al. [31] used graphene oxide as an adsorbent for MO dye adsorption, and the amount adsorbed was equal to 16.83 mg/g, in a contact time of 100 min at pH = 3. Ji et al. [32] evaluated the methyl orange adsorption in organo-vermiculites modified by an asymmetric Gemini surfactant and concluded that the higher amount of modifier implies greater availability of surfactant. In this case, the higher MO dye adsorption measured was equal to 132.03 mg/g. Additionally, it is possible to find published studies about methylene blue in different solvents. Şahin et al. [33] modified the surface of bentonite clay with plasma to improve its adsorptive capacity for MB. Indeed, the plasma treatment increased the adsorptive capacity of bentonite, and the highest value obtained was equal to 303 mg/g. On the other hand, Sellaoui et al. [34] investigated the MB adsorption mechanism on Brazilian berries seeds. Therefore, studies of the MO and MB adsorption in bentonite as an adsorbent are plentiful in the literature. However, few studies are addressed to investigate the adsorption of the anionic dye in acidified calcium bentonites.

On the other hand, the smectites are abundant clay minerals and have a high degree of isomorphic substitutions that generate a deficiency of positive charge and a high capacity of electrostatic interaction [35]. Besides being considered a low-cost adsorbent, it is also easily available, eco-friendly, non-toxic, and has a large surface area, cation exchange capacity, high porosity, and active sites in its surface [36,37,38], which gives it dye adsorption capacity. However, its deficiency of positive charge impairs the adsorption of anionic dyes, even in environments with acid pHs, hampering its applicability in the treatment of waters contaminated with such dyes.

The adsorption mechanism of the calcium bentonites, clays formed by the smectite clay mineral montmorillonite, occurs mainly due to ionic exchanges and electrostatic interactions in the surface of the clay mineral particles [39]. The permanent high negative charge in the clay surface is characteristic of the montmorillonite, which can facilitate adsorption by cationic dyes and impair that of anionic ones [40,41,42]. Thus, changes in the clay structure can be performed to improve the capacity of the adsorption of anionic dyes. The acid treatment shows to be an efficient and low-cost alternative [43] to enhance clay porosity and surface properties [44].

In this respect, this study aims at investigating the efficiency of the acid treatment in Brazilian calcium bentonite from the state of Paraiba for adsorption of the anionic dye methyl orange. The bentonite clay was modified with acids (HCl and H_2_SO_4_), characterized, and had its adsorption capacity evaluated by adsorption tests.

## 2. Materials and Methods

### 2.1. Raw Materials

The calcium bentonite used in this paper derived from the city of Sossego, located in the state of Paraiba–Brazil. The other reagents used were hydrochloric acid P.A (Nuclear), MM: 36.5 g/mol and purity 40%, sulfuric acid P.A (Química Moderna, Barueri-SP, Brazil), MM: 98.08 g/mol and purity 98%, methylene blue P.A (Synth), and methyl orange P.A (NEON).

### 2.2. Acid-Functionalization of Bentonite

Under constant stirring and temperature (90 °C), 25 g of clay were immersed in 250 mL of solutions of the HCl (13.04 mol/L) and H_2_SO_4_ (18.38 mol/L) (4N), in a reflux system. After 2 h, the system was filtered, and the solid was washed repeatedly with distilled water, and then filtered again under vacuum. The filtrate obtained was dried for 48 h at 60 °C [45]. The clays without acid attack were identified as RF, and those activated in acid solutions of HCl and H_2_SO_4_ were identified as RF1 and RF2, respectively.

### 2.3. Characterization

The chemical analysis was determined via X-ray fluorescence (Shimadzu, EDX720, Kyoto, Japan). X-ray diffraction was performed using a Shimadzu XRD6000 (CuKα, 40kV/30mA). All the experiments were performed at room temperature, with a goniometer speed of 2°/min, an angular step of 0.02, and a counting time 0.6 s [46,47,48]. The granulometric analysis was performed by low-angle laser light scattering (Cilas, 1064LD, Orléans, France), where the clays were scattered in 250 mL of distilled water in a Hamilton Beach N5000 stirrer at a speed of 17,000 rpm for 10 min; then, the scatterings were inserted in the equipment in wet mode until they reached the optimal concentration of 150 units of diffraction/incidence area [49,50]. The experiments of differential thermal analysis (BP engineering) and thermal gravimetry (DTG-60H Simultaneous DTA-TG Apparatus Shimadzu) were performed under the air atmosphere using alumina crucibles. All the samples were heated at 12.5 °C/min from room temperature to 1000 °C [51].

### 2.4. Adsorption Experiments

Solutions of 200 mL containing the dyes methyl orange (MO) and methylene blue (MB) were prepared in pre-defined concentrations (25, 50, 75, 100, 125, 150, 200, 250, 300, 350,400 mg/L for MO; and 25, 50, 75, 100, 125, 150, 200, 250 mg/L for MB). For each solution, 100 mg of the RF, RF1 and RF2 clays were added. The adsorption of MO and MB was conducted in aqueous solution keeping the pH = 6, steering of 300 rpm, and temperature (24 °C) constant. The parameters contact time (for MB: 5, 10,15, 30, 45, 60, 90,120 min; and for MO: 15, 30, 45, 60, 90, 120, 180 min) and initial concentration of adsorbate (25 to 400 mg/L for MO, and 25 to 250 mg/L for MB) were investigated.

For each time point studied, 10 mL of solution (clays + dyes) were centrifugated (3600 rpm for 5 min). The solutions were analyzed by UV-VIS spectroscopy (Shimadzu, model UV-1800). The amounts adsorbed by adsorbent mass were evaluated via Equation (1):

(1) $$Q=\frac{\left(C_{i}-C_{e}\right)\cdot V}{m}$$

where *V* is the solution volume (L), m is the adsorbent mass (g), *C_i_* and *C_e_* are the initial and equilibrium concentrations (mg/L), respectively.

### 2.5. Isothermal and Kinetic Studies

The Langmuir [52] (Equation (2)), Freundlich [53] (Equation (3)), Dubinin-Astakhov (D-A) [54] (Equation (4)), Dubinin-Radushkevich (D-R) [55] (Equation (5)), and Sips [30] (Equation (6)) equations were adjusted to the adsorption isotherms to infer surface properties of the natural and acidified bentonite with anionic and cationic dyes. The equations of the isothermic models mentioned are summarized below:

(2) $$Q_{eL}=\frac{Q_{max}^{cal}\;\cdot \;K_{L}\cdot C_{e}}{1+K_{L}\cdot C_{e}}$$

(3) $$Q_{eF}=K_{F}\;\cdot \;{\left(Ce\right)}^{\frac{1}{n_{F}}}$$

(4) $$Q_{eDA}=Q_{max}^{cal}exp\left[-{\left(\frac{RTln\left(C_{s}/C_{e}\right)}{E_{DA}}\right)}^{n_{DA}}\right]$$

(5) $$Q_{eDR}=Q_{max}^{cal}exp\left[-{\left(\frac{RTln\left(C_{s}/C_{e}\right)}{E_{DR}}\right)}^{2}\right]$$

(6) $$Q_{eSIPS}=\frac{Q_{max}^{cal}K_{S}C_{e}^{nS}}{1+K_{S}C_{e}^{nS}}$$

where for the Langmuir equation, $Q_{max}^{cal}$ (mg/g) is the maximum adsorption capacity, *C_e_* (mg/g) is the equilibrium concentration, and *K_L_* (L/mg) is the Langmuir constant. In the Freundlich equation, *K_F_* (mg^1−(1/n)^g^−1^L^1/n^) is the Freundlich adsorption capacity constant, and n_F_ is an empirical constant indicating adsorption intensity. In the D-A and D-R models, $Q_{max}^{cal}$ has the same meaning as that observed in the Langmuir equation, *n_DA_* is the adjustment parameter proposed by Dubinin-Astakhov, ε (KJ/mol) is the potential for adsorption, and *E_DA_* (KJ/mol) is the characteristic energy of the system. *C_S_* and *C_e_* are solubility and equilibrium concentration, respectively. *E_DR_* (KJ/mol) is the parameter for estimating the free energy of the system. In Equation (6), *K_S_* is the Sips isothermal constant (L/mmol), and *n_S_* indicates the system heterogeneity.

The pseudo-first-order [56] and pseudo-second-order models [57] were used to infer adsorption kinetics between the MB and MO dyes and RF, RF1 and RF2 clays, see Equations (7) and (8):

(7) $$Q_{tPFO}=Q_{e}\;\left(1-e^{-K_{1}\cdot t}\right)$$

(8) $$Q_{tPSO}=\frac{K_{2}\;\cdot Q_{e}^{2}\cdot t}{1+K_{2}\cdot Q_{e}\cdot t}$$

In Equations (7) and (8), *Q_t_* (mg/g) and *Q_e_* (mg/g) are the amount of dyes adsorbed in time *t* (min) and at equilibrium, respectively; *K*_1_ and *K*_2_ are the speed constants of pseudo-first-order (min^−1^) and pseudo-second-order (g.mg.min^−1^), respectively.

## 3. Results and Discussion

### 3.1. Characterization of Natural and Activated Clays

Table 1 shows the chemical analysis obtained from the RF, RF1, and RF2 clays. As expected, SiO_2_ and Al_2_O_3_ were the major mineral constituents both for natural clay and for clays activated with the acids HCl and H_2_SO_4_. The components MgO and Fe_2_O_3_ were also identified in small amounts in the RF, RF1 and RF2 clays; however, the CaO was identified in the RF and RF_1_ clays only. It is well known that Ca^2+^ ions are present in the interlamellar layer, and the Mg^2+^ and Fe^3+^ ions are present in the octahedral sheets [58]. These three constituents (CaO, MgO, and Fe_2_O_3_) negatively influence the adsorbent capacity of calcium bentonite because cationic exchanges can be prejudiced. In this regard, the acid attack can increase the adsorbent capacity of calcium bentonite, once the Ca^2+^, Mg^2+^ and Fe^3+^ ions are decreased (or completely removed). The other oxides were identified to a lesser degree, with percentage values lower than 0.5%.

Additionally, in Table 1, the increase in the amount of SiO_2_, upon acid attack, is related to the partial destruction of the octahedral layers and the reduction of the components Al_2_O_3_, Fe_2_O_3_, MgO and CaO in the samples treated with acid. The Mg^2+^, Fe^3+^ and Ca^2+^ ions have a high rate of dissolution in the HCl and H_2_SO_4_ acids, being this rate higher in H_2_SO_4_ [59]. For this reason, the decrease of such ions was more marked in the sample RF2. In the sample RF2, the attack with H_2_SO_4_ thoroughly removed the CaO. This behavior is related to the fact that the Ca^2+^ ions in polycationic clays are easily dissolved by the protons, due to being located in the interlamellar space of calcium bentonite [60]. Additionally, potential carbonates and calcite are also naturally dissolved in acid environments.

**Figure 1:**
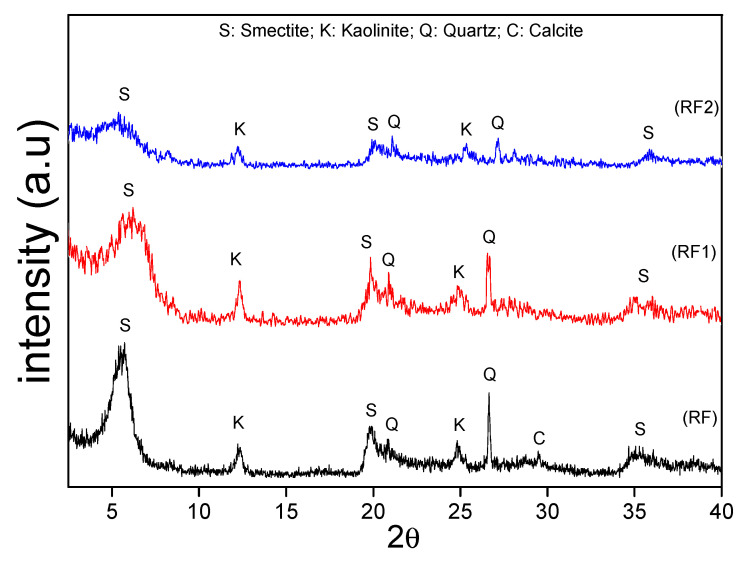
X-ray diffractograms obtained from the RF, RF1, and RF2 clays.

Figure 1 shows diffractograms obtained from the samples RF, RF1, and RF2. The crystalline phases identified were smectite (JCPDS 13-0135), quartz (JCPDS 46-1045), kaolinite (JCPDS 78-2110), and calcite (JCPDS 84-0710). Based on the qualitative analysis, we can note that the acid treatment resulted in a decrease in the intensity of peaks in the samples RF1 and RF2 [60]. The decreased intensity of the montmorillonite main peak indicates that there was a partial destruction and rearrangement of the ions in the tetrahedral and octahedral layers of the clays [61,62,63].

**Figure 2:**
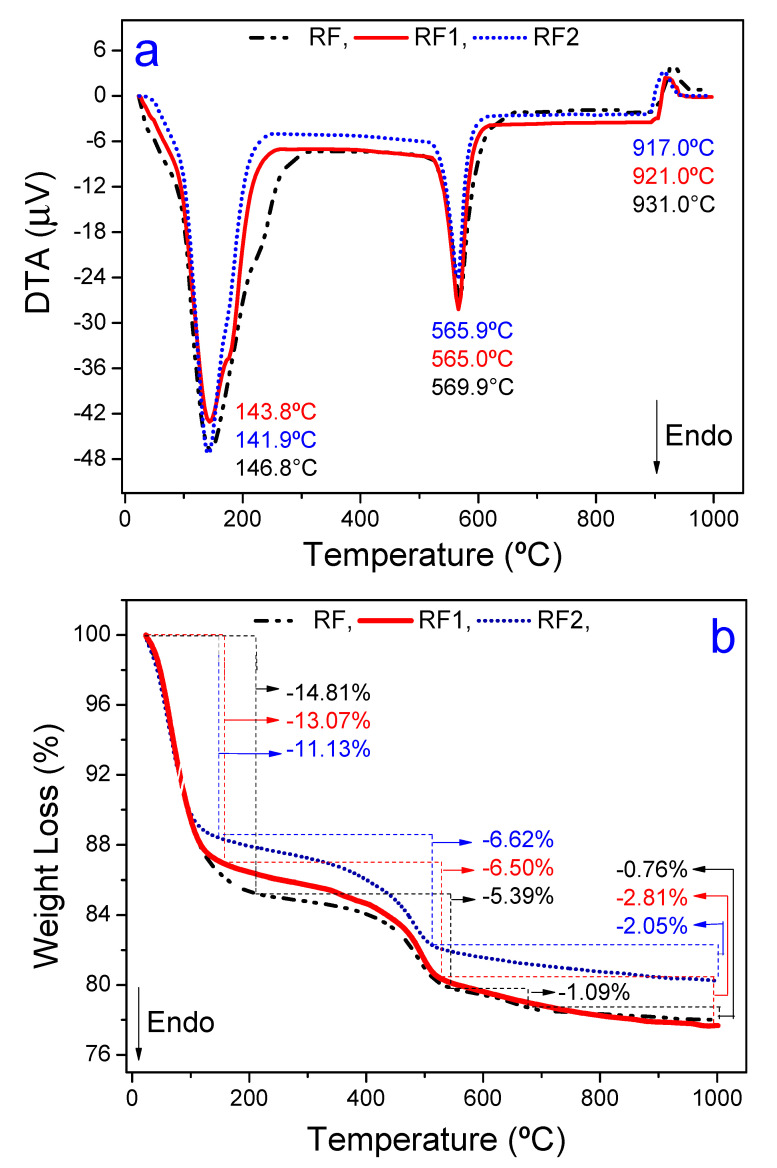
Shows DTA (**a**) and TG (**b**) curves measured from the RF, RF1, and RF2 clays.

Figure 2a,b shows the DTA and TG curves of the RF, RF1, and RF2 clays. Figure 2a shows the DTA curves; in general, the curves show three thermal events. The first thermal event has an endothermic nature, and the peak maximum temperatures were 146.8 °C, 143.8 °C, and 141.8 °C for the RF, RF1 and RF2 clays, respectively. This first event is related to the evaporation of water (free, adsorbed, and coordinated) [64]. Still, on the first thermal event, we can see a shoulder (more evident in the RF clay, 200 °C to 280 °C), which is often associated with polycationic bentonite with more than one exchangeable cation, such as calcium and magnesium, [65]. The second event was endothermic as well and is related to the partial dehydroxylation of the octahedral sheet of smectite, and the peak maximum temperatures were 569.9 °C, 565.0 °C and 565.9 °C for the RF, RF1 e RF2 clays, respectively. The third thermal event was exothermic and is related to mullite nucleation [66], and the peak maximum temperatures were 930.0 °C, 921.0 °C, and 917.0 °C for the RF, RF1 e RF2 clays, respectively. There were no significant differences in the intensities of the thermal events in Figure 2a. This behavior indicates that acid treatment did not cause expressive destruction in the crystalline reticulate of the clay minerals found in bentonite. However, the acid activation enabled protonation in the structure of the material, creating acid sites and removing impurities with partial destruction of the crystalline reticulate through dehydroxylation of the structural hydroxyl groups present in the octahedral layer of the clay [67,68].

Figure 2b shows TG curves in which losses of mass could be found concerning the thermal events showed in the DTA curves (see Figure 2a). Therefore, the most marked losses of mass occurred in the first thermal event, and they were 14.81%, 13.07%, and 11.13% for RF, RF1, and RF2, respectively. This behavior indicates that the hydroxyl groups are leaving more efficiently [43]. The losses of mass related to the second thermal event were 5.39%, 6.62% and 6.50% for the RF, RF1 and RF2 clays, respectively. For the RF clay only, a mass loss of 1.09% was identified at ~648 °C, which is related to the calcium carbonate decomposition. This thermal event was not seen in the other clays (RF1 and RF2), because the acid attack significantly removed the amount of CaO (0.22 wt% and 0.0 wt. % for the RF1 and RF2 clays, respectively). In the last thermal event, the mass losses were 0.76%, 2.05%, and 2.81% for the RF, RF1, and RF2 samples. The total mass losses measured were 22.05%, 22.38%, and 19.80% for the RF, RF1, and RF2 clays, respectively.

**Figure 3:**
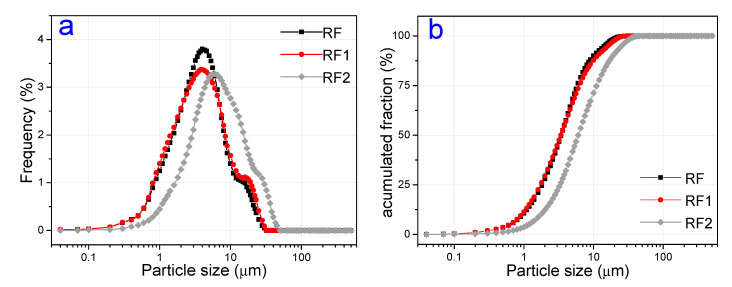
Granulometric analysis of the clays: (**a**) Frequency, and (**b**) cumulative values.

Figure 3 shows the size distribution of the particle of the RF, RF1, and RF2 clays. All the curves showed a bimodal behavior, in which the major concentration of particles was seen between 2 μm and 20 μm. The RF1 sample showed a higher fraction of particles with a diameter smaller than 2 μm when compared to the RF and RF2 samples. In general, the largest average diameter of particles was seen for the samples with acid activation, results also observed by [67,69]. This behavior can be related to the likely increase in material porosity after acid activation and to the creation of specific charge sites in the clay surface that favor the electrostatic attraction between the small particles of the clays, generating small agglomerates, which are observed as large particles in the particle size determination test. The lower fraction of particles with a small diameter than 2 μm of RF2 can be related to this charge characteristic [70,71]. Table 2 summarizes the granulometric compositions and average size of particles.

### 3.2. Adsorption Tests

**Figure 4:**
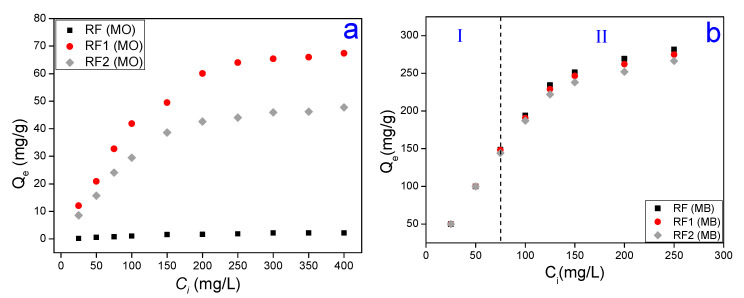
The amounts adsorbed of the MO (**a**) and MB (**b**) dyes for different initial concentrations of the adsorbate.

Figure 4a,b shows the adsorbed amount dependency of the MO and MB dyes due to different initial concentrations of the adsorbate. In general, it was noted that the amount adsorbed ($Q_{e}$) of MO and MB increases as the initial concentration increases [43]. At a molecular scale, this behavior occurs because the $C_{i}$ increase causes more interactions between the clay surface and the dye molecules, implying a rise in the adsorption rate [72]. Additionally, the increased adsorption rate can be explained due to the increase of charge of the adsorbent in terms of the elevated driving force to transfer the existing mass in greater concentrations of dye [73]. In Figure 4a, an increase in $Q_{e}$ can be seen for the clays treated with acid. Indeed, for the MO dye, the acid activation increased the adsorption capacity of the clays in up to 8000%, with increased value of 2.2 mg/g in the RF, for 67.4 mg/g in the RF1 and 47.8 mg/g in the RF2. It is known that the calcium bentonite, such as the other clays, has a negative charge in its surface structure that derives from isomorphic substitutions of the Al^3+^ for Si^4+^ ions in the tetrahedral layer, and Mg^2+^ for Al^3+^ ions in the octahedral layer [74]. For this reason, the maximal MO adsorption in RF was insufficient due to the electrostatic repulsion between the negative charge in the clay surface and the negative nature of the MO.

The acid attack creates Brönsted acid sites (positive nature) in the clay surface that are generated by the exchange of cations existing in interlamellar layers (Ca^2+^ and Mg^2+^) by protons derived from the acid [67], and these sites created attract methyl orange molecules (anionic nature) more efficiently and exchange reactions occur. Another effect that also occurs is the removal of Al^3+^ ions from the octahedral layers and the increase of the clay mineral surface area [43]. These behaviors can be ratified by the decrease of the CaO, MgO, and Al_2_O_3_ components upon acid attack (see Table 1).

Figure 4b can be divided into two regions, where region I is included in the rate of 25 mg/L and 75 mg/L of the $C_{i}$. In this region, both the natural clay (RF) and the clays activated with HCl and H_2_SO_4_ acids (RF1 and RF2, respectively) adsorbed the same amount of the MB dye. As to region II, consisting of the rates 76 mg/L and 250 mg/L of $C_{i}$, it was noted that for the RF1 and RF2 clays the $Q_{e}$ values were slightly lower than for the RF clay, having $Q_{max}^{exp}$ of 281.7 mg/g (RF), 274.7 mg/g (RF1) and 266.3 mg/g (RF2). The MB cationic dye adsorption derives from electrostatic forces arising between the negative charge of the clay and the positive nature of the MB [75]. Therefore, according to the region II (Figure 4b), in the acid-activated clays, as the dye concentration increased, there was also an increase of $Q_{e}$. The slight difference of the amount adsorbed between the acid bentonites and RF, although not statistically representative, can be attributed to the fact of electrostatic interactions are more intense in the natural clay for having more negative sites in its surface.

Comparing Figure 4a,b, for all the $C_{i}$ values, we can see that the $Q_{e}$ values of the MO dye is much higher in the acid-activated bentonite than natural bentonite. As for the MB dye, no significant differences were seen between the amounts adsorbed by the RF, RF1 and RF2 clays. This confirms the fact that the acid treatment is efficient in calcium bentonites for adsorption of anionic dyes. This could be explained in terms of the electrostatic interaction between the clay and adsorbate, once it had been determined that the acid attack generates Brönsted acid sites, which more easily attract the methyl orange molecules (anionic nature), and these new, more active sites repel the cationic nature of dyes, which explains why the acid treatment was not effective on the clay for adsorption of the MB dye, showing statistically similar RF, RF1 and RF2 results.

It is well known that sulfuric acid can generate a high removal of the octahedral Al^3+^ ions, an increase in the Si-OH bonds, and the formation of a high amount of amorphous silica phase [71,76]. These phenomena will favor the development of negative charges on the surface of the clay minerals. The HCl also attacks the structure of the clay mineral but in a lower amount, preventing the natural adsorptive properties of montmorillonite clay from being destroyed. According to Figure 4a, it can be seen that the acid attack was more effective for HCl acid, previous studies [77,78] have already shown that the activity of hydrochloric acid for pollutant adsorption is higher in clays when compared to sulfuric acid in the same normality. This will favor a higher degree of adsorption of the anionic dyes in HCl treated samples.

**Figure 5:**
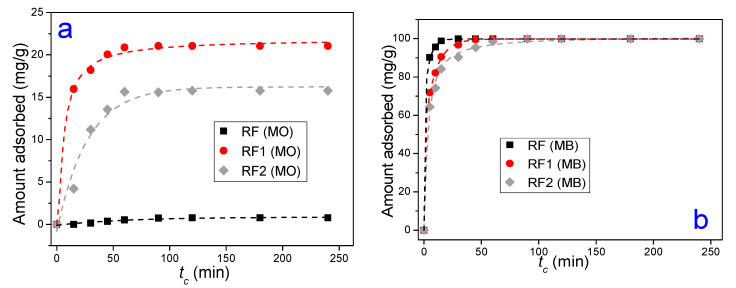
Effect of contact time (*t_c_*) on (**a**) MO adsorption and (**b**) MB adsorption. The lines are only guides for the eyes.

Figure 5a,b relates the adsorbed amounts of the MO and MB dyes in times set ($t_{c}$) in the RF, RF1 and RF2 samples, fixing $C_{i}$ at 50 mg/L. For Figure 5a, the values of $Q_{e}$ obtained for the MO were 2.2 mg/g for RF, 67.4 mg/g for RF1, and 47.8 mg/g for RF2. In general, we can see that the kinetics of the RF clay is different from that shown in the acid ones for the experimental conditions investigated, due to not having interaction between the clay and the MO, which resulted in low $Q_{e}$ values. In RF1 and RF2, there was a rapid increase in the amount of dye adsorbed in the clay at the initial part of the curve, i.e., the kinetics was higher in the activated clays. This occurs because there is a higher concentration of active sites available in the clay during the initial part of the experiment. Over time, there was a decrease in the concentration of sites available until it reaches equilibrium. The equilibrium is represented from the moment when the amount adsorbed did not vary significantly, i.e., the clay surface is saturated by the dye. The equilibrium times measured for the MO dye were 120 min for all the clays studied. As to the MB dye (Figure 5b), the $Q_{e}$ values were the same for all the clays studied at about 100 mg/g, and the kinetic behavior did not vary significantly with the acid treatment in the cationic dye. The equilibrium times were 30 min, 45 min, 90 min for the RF, RF1, and RF2 clays, respectively. The major equilibrium time seen in the acid-activated samples were possibly due to the creation of new active sites in the clay [74]. The equilibrium times found in this work are in accordance with those found in the literature [39,79,80].

**Figure 6:**
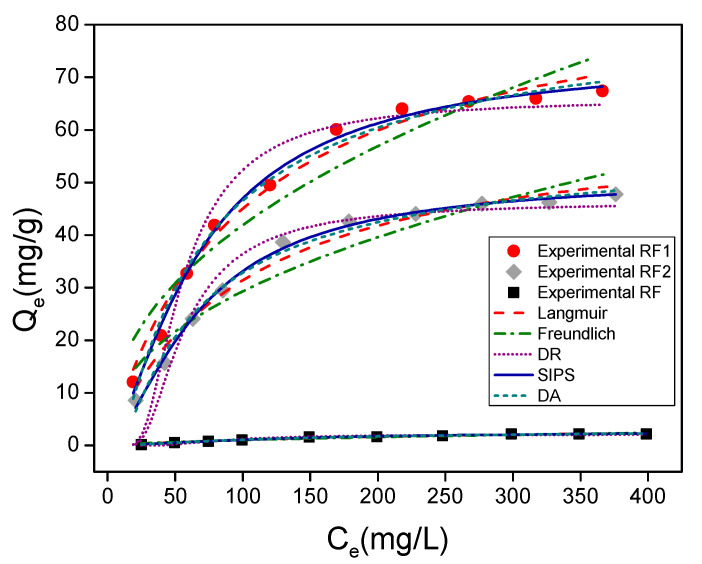
Adsorption isotherms of MO in room temperature conditions, adsorbent mass 100 mg, fixating $t_{c}$ in 120 min.

**Figure 7:**
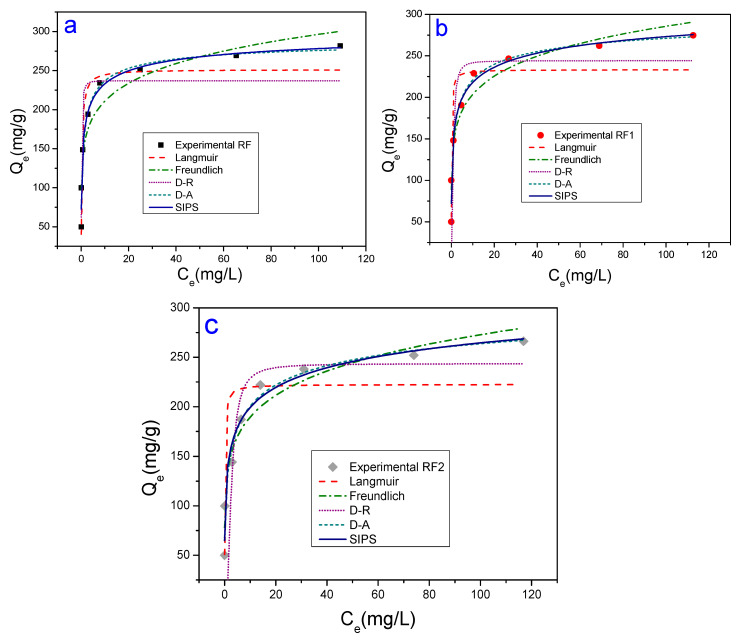
Adsorption isotherms of MB in room temperature conditions, adsorbent mass 100 mg, using $t_{c}$ in 90 min: (**a**) adsorbent RF; (**b**) adsorbent RF1; and (**c**) adsorbent RF2.

Figure 6 and Figure 7a–c show adsorption isotherms obtained for the MO and MB dyes, respectively. For the MO dye (Figure 6), the adsorbent amount and $t_{c}$ were 100 mg and 120 min, respectively. For the MB dye (Figure 7), the amount of adsorbent used was equal to 100 mg for $t_{c}$ of 90 min, respectively. All the experiments were conducted at room temperature. The $Q_{max}^{calc}$ values, the n parameters (n_F_, n_DA_, n_S_), and adsorption capacity constants (K_L_, K_F,_ K_DR_, K_DA_ e K_S_) were evaluated by the Langmuir, Freundlich, Dubinin-Radushkevich, Dubinin-Astakhov, and Sips models.

The results of the mathematical adjustments implemented in Figure 6 and Figure 7a–c are summarized in Table 3. From the MO experimental data, it was possible to see that the best correlation coefficients were obtained from Sips and D-A equations (acid clays: R^2^ > 0.99; natural clay: R^2^ > 0.98). The $Q_{max}^{calc}$ values of the MO adsorbates, inferred from the Sips equation, were closer to those measured experimentally in Figure 6. As previously mentioned, the $Q_{max}^{exp}$ values measured in this work were 2.2 mg/g, 67.4 mg/g and 47.8 mg/g for the RF, RF1 and RF2 clays, respectively. Additionally, the error values are also lower for the Sips equation, indicating that this is the modeling that best describes the process of adsorption for MO in these adsorbents. Related the cationic dye studied (methylene blue), the results of Table 3 are in agreement with the experimental results in Figure 7a–c since both showed that the adsorption capacity did not change significantly, i.e., the acid attack was not efficient for improving the sorption of the cationic dye in the calcium bentonites investigated.

The Sips equation is based on the theory that at low adsorbate concentrations, the adsorption is described by the Freundlich equation. At high adsorbate concentrations, the adsorption is already characterized by the Langmuir equation; that is, it occurs via monolayers [81]. The K_S_ adsorption capacity constant obtained from the Sips equation is in agreement with Figure 6 since acid-functionalized calcium bentonite presented higher K_S_ values (7.7 × 10^−4^, 2.1 × 10^−3^, and 1.5 × 10^−3^ to RF, RF1, and RF2, respectively). On the other hand, the 1/n_S_ values, also acquired from the Sips equation, varied between 0.68 and 0.64. It is known that 1/n_S_ values less than 1, indicating a certain degree of heterogeneity on the surface of the acid-functionalized bentonite [82]. The heterogeneous surface on the acid-functionalized clay occurs because the acid attack disorganizes its structure favoring an increase in the surface area, with consequent formation of mesopores and ions leaching (Ca^2+^ and Mg^2+^, in the case of this work) [83,84]. The experimental evidence for the leaching of Ca^2+^ and Mg^2+^ ions can be seen from the analysis in Table 1.

Additionally, the presence of H^+^ ions resulting from the acid protonated the clay surface. Due to this, the adsorption of anionic dye on the acid-functionalized bentonite is likely to occur initially on its surface (more reactive) by an exchange reaction until dye molecules occupy all available sites. Subsequently, the molecules diffuse to the clay layers, continuing the adsorption process through ion exchange reactions. This process suggests the formation of multilayers [85,86].

**Figure 8:**
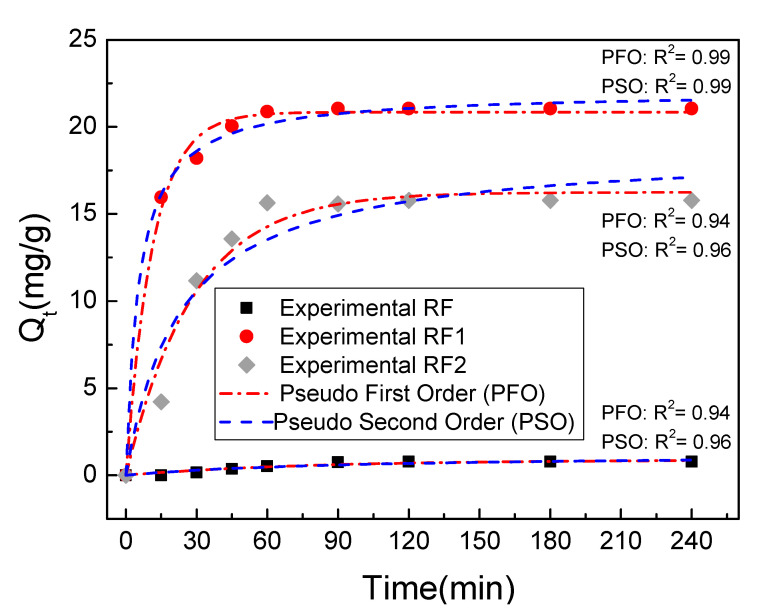
Kinetic graph of MO in room temperature conditions, adsorbent mass 100 mg, fixating (C_i_) in 50 mg/L.

**Figure 9:**
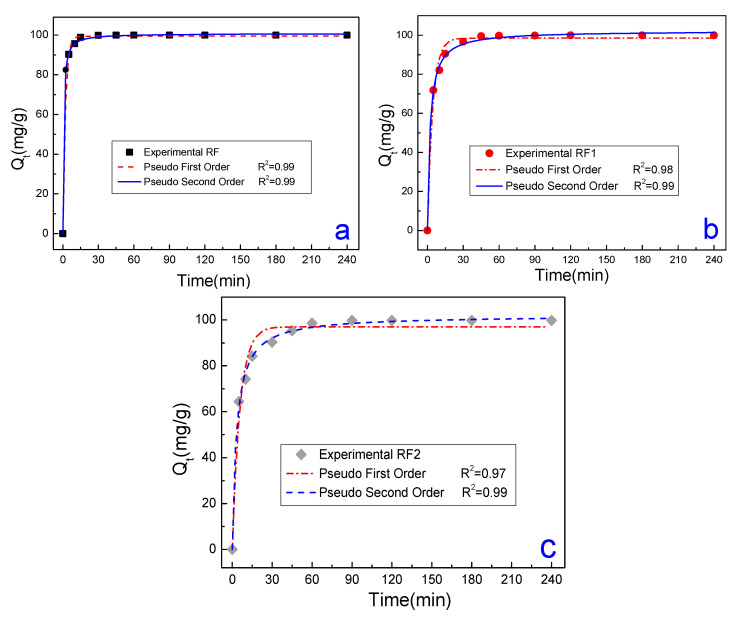
Kinetic graphs of MB in room temperature conditions, adsorbent mass 100 mg, fixating Ci in 50 mg/L: (**a**) adsorbent RF; (**b**) adsorbent RF1; and (**c**) adsorbent RF2.

The adsorption mechanism of the MO and MB dyes to the RF, RF1, and RF2 clays were also investigated from mathematical adjustments performed with the aid of pseudo-first-order and second-order models, Figure 8 and Figure 9. All of the adjustments performed were satisfactory, and the results are summarized in Table 4.

From Table 4, we can see that both models, first- and second-order, quantitatively described the $Q_{e}$ values compared the Q_1_ (first-order equation) and Q_2_ (second-order equation) values with the $Q_{e}$ values of the MO and MB dyes showed in Figure 8 and Figure 9. However, the pseudo-second-order model showed better R^2^ values (near 1), indicating a better description of the adsorption kinetics. From the concept of this model, it is possible to infer that the limitation rate of the dye molecules adsorption in the clay occurs mainly through chemisorption [36,37,86,87]. Therefore, the second-order model is the most indicated to describe the adsorption mechanism, possibly due also to the interaction between the dye molecules and the new sites generated on the surface.

**Figure 10:**
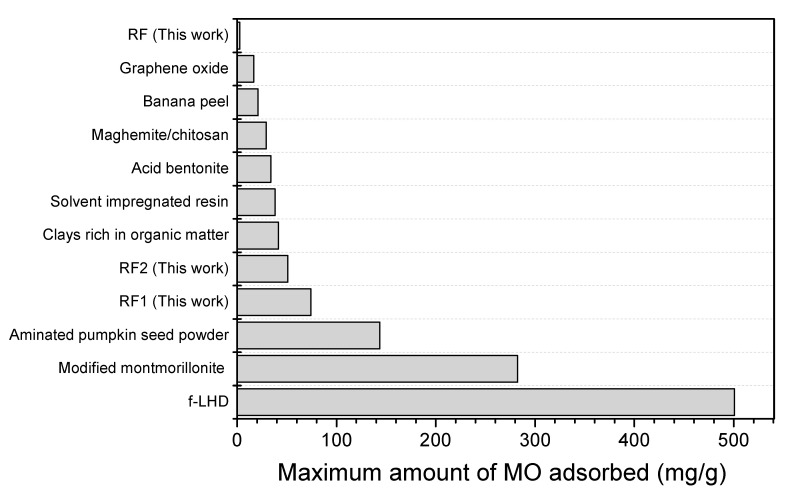
Comparison of $Q_{max}^{calc}$ of the MO dye for different adsorbents studied in the literature.

Figure 10 compares $Q_{max}^{calc}$ of the MO adsorbed by the RF, RF1 and RF2 clays with the $Q_{max}^{cal}$ values by other adsorbents found in the literature. The mathematical adjustment performed by the Sips equation conducted at the MO adsorption isotherm was satisfactory: R^2^ = 0.987 in RF and for the activated R^2^ = 0.993 and R^2^ = 0.995 for RF1 and RF2, respectively. Therefore, the maximal adsorbed amounts of MO used to construct Figure 10 are those shown in Table 3.

In this regard, Figure 10 compares the $Q_{max}^{calc}$ of MO obtained in this paper with literary data for graphene oxide [31], acid bentonite [88], solvent impregnated resin [89], clays rich in an organic matter [90], banana peel [91], maghemite/chitosan [92], aminated pumpkin seed powder [93], montmorillonite modified by gemini surfactants [94], and layered double hydroxides with flower-like morphology (f-LHD) [95]. In our paper, the RF clay showed the worst $Q_{max}^{calc}$ of MO dye. As discussed before, this is because the natural clay has a predominantly negative contact surface while the MO dye is anionic. The acid attack applied $Q_{max}^{calc}$ of MO (RF1 and RF2) satisfactorily, as it increased the adsorption capacity of the anionic dye and also showed better values compared to those investigated by [31,88,89,90,96]. The adsorbents aminated pumpkin seed powder [93], montmorillonite modified by gemini surfactants [94] and layered double hydroxides with flower-like morphology (f-LHD) [95], showed values of 143.7 mg/g, 282.4 mg/g, and 500.6 mg/g, respectively. The authors used adsorbents modified with more complex and expensive techniques that can be difficult to apply, in contrast to that used in this paper.

## 4. Conclusions

The purpose of this paper was to evaluate the efficiency of the acid treatment in Brazilian bentonite for the adsorption of anionic dye methyl orange. The attack with acids did not significantly destructed the structure of the clay minerals found in the clay, with a decrease in the concentration of Ca^2+^ Mg^2+^ and Fe^3+^ ions. It was possible to raise the adsorption capacity of 2.2 mg/g to 67.4 mg/g of methyl orange with acid treatment, which is an increase of 8000% in anionic dye adsorption. The acid activation did not significantly change the clay adsorption capacity in relation to the cationic dye used as a probe, the methylene blue, with the acid clay reaching values of 274.7 mg/g (RF1) and 266.3 mg/g (RF2). The bentonite has adsorption based on a heterogeneous surface. Acid activation of the bentonite maximized the adsorption capacity of the anionic dye. However, it did not significantly reduce the adsorption capacity of the cationic dye, making the clay a potentially commercial, sustainable, and eco-friendly material for the treatment of waters contaminated with dyes.

## Figures and Tables

**Table 1:** Chemical composition (wt%) of the samples RF, RF1, and RF2.

Clays	SiO_2_	Al_2_O_3_	Fe_2_O_3_	MgO	CaO	TiO_2_	RuO_2_	K_2_O	Cr_2_O_3_	SO_3_	Others	LF *
RF	45.3	20.7	5.9	2.7	1.6	0.8	0.2	0.5	0.04	0.1	0.05	22.1
RF1	47.8	20.5	5.7	2.2	0.2	0.8	-	-	0.03	0.1	0.03	22.3
RF2	54.2	19.1	3.3	1.7	-	0.5	-	-	-	1.1	0.42	19.7

* LF: loss of fire.

**Table 2:** Granulometric compositions and average sizes of particles measured from the RF, RF1, and RF2 samples.

Samples	D < 2 μm	(2 μm < D < 20 μm)	D > 20 μm	Average Diameter (μm)
RF	27.98%	70.77%	1.25%	4.66%
RF1	29.55%	68.31%	2.14%	4.96%
RF2	12.31%	75.37%	8.92%	8.46%

**Table 3:** Parameters of MO and MB adsorption isotherms.

Models	Parameters	Methyl Orange			Methylene Blue		
		RF	RF1	RF2	RF	RF1	RF2
Langmuir	$Q_{\mathrm{cal}}^{\max}$(mg/g) Error	3.84	89.48	62.34	251.4	233.3	222.5
		0.41	4.54	3.01	16.57	17.31	16.46
	K_L_(L/mg)	3.8 × 10^−3^	1.0 × 10^−2^	1.0 × 10^−2^	3.62	8.89	7.98
	Error	7.9 × 10^−4^	1.4 × 10^−3^	1.3 × 10^−2^	1.86	5.64	4.86
	R^2^	0.975	0.976	0.977	0.840	0.761	0.753
Freundlich	K_F_ (mg^1−(1/n)^g^−1^L^1/n^)	5.9 × 10^−2^	5.49	3.97	150.23	145.05	131.73
	Error	2.2 × 10^−2^	1.74	1.26	11.78	9.85	9.11
	n_F_	1.61	2.26	2.30	6.77	6.80	6.32
	Error	0.07	0.30	0.32	1.01	0.87	0.76
	R^2^	0.945	0.908	0.904	0.904	0.933	0.950
D-R	$Q_{\mathrm{cal}}^{\max}$(mg/g)	2.15	65.92	46.31	237.03	244.11	243.47
	Error	0.11	2.61	1.71	17.22	23.71	23.78
	K_DR_ (KJ/mol)	9.2 × 10^−4^	3.8 × 10^−4^	3.9 × 10^−4^	2.4 × 10^−8^	1.8 × 10^−−7^	1.1 × 10^−6^
	Error	1.7 × 10^−4^	6.5 × 10^−5^	6.6 × 10^−5^	8.4 × 10^−9^	1.3 × 10^−7^	6.9 × 10^−7^
	R^2^	0.928	0.926	0.932	0.768	0.602	0.617
D-A	$Q_{\mathrm{cal}}^{\max}$(mg/g)	3.51	86.40	58.14	294.24	311.19	347.67
	Error	0.70	7.86	4.09	22.64	38.56	74.33
	K_DA_ (KJ/mol)	6.78	4.45	4.92	0.01	0.03	0.10
	Error	1.66	0.73	0.82	0.01	0.05	0.10
	n_DA_	0.71	0.80	0.86	0.53	0.40	0.30
	Error	0.17	0.13	0.12	0.12	0.11	0.10
	R^2^	0.985	0.986	0.988	0.964	0.962	0.961
Sips	$Q_{\mathrm{cal}}^{\max}$(mg/g)	2.68	74.23	51.10	314.02	353.42	403.37
	Error	0.25	2.58	1.23	33.19	72.73	139.19
	K_S_(L/mmol)	7.7 × 10^−4^	2.1 × 10^−3^	1.5 × 10^−3^	1.07	0.76	0.50
	Error	6.8 × 10^−4^	1.0 × 10^−3^	6.1 × 10^−4^	0.32	0.33	0.28
	n_S_	1.47	1.45	1.54	0.43	0.33	0.29
	Error	0.22	0.13	0.10	0.09	0.08	0.08
	R^2^	0.987	0.993	0.995	0.985	0.981	0.987

**Table 4:** Kinetic parameters of MO and MB adsorption.

Models	Parameters	Methyl Orange			Methylene Blue		
		RF	RF1	RF2	RF	RF1	RF2
Pseudo-first-order	Q_1_ (mg/g)	0.88	20.85	16.25	99.50	98.51	97.03
	Error	0.11	0.23	0.63	0.36	1.21	1.79
	K_1_ (min^−1^)	0.01	0.09	0.04	0.46	0.23	0.17
	Error	0.01	0.01	0.01	0.02	0.02	0.02
	R^2^	0.94	0.99	0.94	0.99	0.98	0.97
Pseudo-second-order	Q_2_ (mg/g)	1.23	22.02	18.76	100.80	102.28	102.03
	Error	0.27	0.26	1.53	0.30	0.59	0.76
	K_2_(gmin^−1^min^−1^)	8.3 × 10^−3^	8.3 × 10^−3^	2.3 × 10^−3^	18.4 × 10^−3^	9.6 × 10^−3^	9.0 × 10^−3^
	Error	5.8 × 10^−3^	1.0 × 10^−3^	9.2 × 10^−4^	1.61 × 10^−3^	2.8 × 10^−4^	1.9 × 10^−4^
	R^2^	0.96	0.99	0.96	0.99	0.99	0.99
